# Learning curve and global benchmark values of laparoscopic sleeve gastrectomy: results of first 100 cases of a newly trained surgeon in an Italian center of excellence

**DOI:** 10.1007/s13304-021-01121-4

**Published:** 2021-06-29

**Authors:** Antonio Vitiello, Giovanna Berardi, Nunzio Velotti, Vincenzo Schiavone, Mario Musella

**Affiliations:** Advanced Biomedical Sciences Department, Naples “Federico II” University, AOU “Federico II”, Via S. Pansini 5, 80131 Naples, Italy

**Keywords:** Sleeve gastrectomy, Surgical training, Learning curve, Global benchmarks, Complications

## Abstract

To evaluate whether the learning curve for sleeve gastrectomy could be completed after 50 cases. First 100 patients undergoing LSG under a newly trained laparoscopic surgeon were included in this study and divided into two groups of 50 consecutive patients each. Perioperative outcomes were compared to recently introduced global benchmarks. Short-term weight loss was calculated as Total Weight Loss Percent (%TWL) and complications were classified in accordance with the Clavien–Dindo classification. CUSUM analysis was performed for operative time and hospital stay. Mean preoperative age and BMI were 41.8 ± 10.3 years and 42.9 ± 5.4 kg/m^2^, respectively. Demographics and rate of patients with previous surgery were comparable preoperatively in the two groups. Mean operative time was 92.1 ± 19.3 min and hospital stay was 3.4 ± 0.6 days as per our standard protocol of discharge. Uneventful postoperative course was recorded in 93% of patients and only one case of staple line leak was registered in the first 50 cases (group 1). No statistical difference in BMI and %TWL was found between the two groups at any time of follow-up. Comparison between two groups showed a significant reduction in hospital stay and operative time after the first 50 LSGs (*p* < 0.05). LSG can be performed by newly trained surgeons proctored by senior tutors**.** At least 50 cases are needed to meet global benchmark cut-offs and few more cases may be required to reach the plateau of the learning curve.

## Introduction

Laparoscopic sleeve gastrectomy (LSG) is currently the most commonly performed procedure worldwide [1] and main reason of its rapid success is the laparoscopic feasibility [2]. LSG was initially introduced by Marceau [3] as first part of the duodenal switch operation to preserve vagal innervation and pyloric function. Subsequently, Gagner [4] proposed a staged procedure also for gastric bypass and LSG was the first step, mostly to overcome the challenge of laparoscopic bariatric surgery in patients with BMI > 60 kg/m^2^. Since postoperative outcomes demonstrated low morbidity and satisfactory weight loss, LSG achieved the status of a stand-alone intervention [5].

Despite this feasibility, even for LSG an appropriate learning curve (LC) is mandatory for newly trained surgeons to reduce perioperative complications [6, 7]. Previous studies have proposed a minimum number of 100 cases to reach a significant reduction in operative time and morbidity after Roux-en-Y Gastric Bypass (RYGB) [8, 9], while a shorter LC has been reported for LSG [10, 11]. However, the precise number of LSGs required to achieve optimal results is still matter of debate.

Recently, global benchmarks for LSG and RYGB were set as the 75th percentile of morbidity in 19 high-volume academic centres in 3 continents: below this value perioperative outcomes are considered acceptable [12].

Aim of this study was to evaluate whether a newly trained laparoscopic surgeon can complete the LC for LSG after 50 cases by comparing perioperative outcomes to recently introduced global benchmarks.

## Methods

Data of first 100 patients undergoing LSG under a newly trained laparoscopic surgeon at our teaching university hospital (caseload > 150 per year; annual number of publications > 4) were retrospectively collected and included in this study.

Indications for surgery followed the recommendations of the International Federation of Surgery for Obesity (IFSO) [13].

Since the main inclusion criteria was the chronological order, also patients with a body mass index (BMI) > 50 kg/m^2^ or with a previous history of bariatric or abdominal surgery were included.

All the procedures were performed by the same surgeon who had recently (< 1 year) ended his residency in general surgery. LC of the novel surgeon was assessed using the CUSUM analysis and divided in two groups (Group 1 and 2) of 50 consecutive patients each and perioperative outcomes were compared to the abovementioned global benchmarks.

Data on preoperative demographics (gender, age, related comorbidities, body mass index—BMI and history of previous bariatric surgery, number of superobese subjects), peri-operative data (operative time, conversion to open, use of staple-line reinforcement, reoperation rate, length of hospital stay, readmissions, intra- and post-operative complications, mortality) were registered. Weight loss was calculated at 1, 3 and 6 months as change of BMI and percentage of total weight loss (%TWL) using the following formula:

$$ \left[ {{\text{initial weight}} - {\text{final weight}}/{\text{initial weight}}} \right] \times {\text{1}}00. $$

Postoperative complications were classified in accordance with the Clavien–Dindo classification [14].

Cases requiring supervision from a senior expert surgeon were also recorded and compared.

The present research was approved by the institutional review board of our Department and informed consent was obtained from all patients before surgery.

### Surgical technique

Standard technique for LSG has been previously reported [15–17]: a five trocars approach (3 × 12 mm, 2 × 5 mm) was used. The gastrectomy started 2–4 cm from the pylorus over a 38–40 French bougie. Staple line reinforcements or oversewing are not routinely used at our Institution. The nasogastric tube was removed the day after surgery and an abdominal drain was left along the staple line. A liquid diet was started on postoperative day 3 and discharge was scheduled in case of no clinical signs of leak or stenosis.

### Statistical analysis

Data are expressed as mean ± DS. Two-tailored *t* test was used to compare continuous variables as appropriate, while categorical data were compared using the Chi-square or Fisher’s exact test. Significant *p* value was set below 0.05.

A cumulative sum (CUSUM) analysis was performed for operative time and hospital stay [18, 19]. Predefined limits were set. Results were presented in CUSUM charts which are a graphical presentation of the outcomes of a series of consecutive procedures. During the LC, the CUSUM curve runs above a decision interval when an operation is performed at an unacceptable level. The intervals were set according to global benchmark values (duration of the operation = 90 min; hospital stay = 3).

## Results

### Baseline characteristics

One hundred consecutive patients were included in the present study; female/male ratio was 16/84 and mean preoperative age and BMI were 41.8 ± 10.3 years and 42.9 ± 5.4 kg/m^2^ respectively. Eight subjects had previously undergone a bariatric procedure (gastric band) and 18 had a precedent history of abdominal surgery (10 caesarean sections, 4 laparoscopic cholecystectomy, 2 umbilical hernia repairs, 2 appendectomy). Demographics and rate of patients with antecedent surgery were comparable preoperatively in the two groups (Table 1).

**Table 1:** Comparison of demographics in the two groups of patients

Parameter	Group 1 (*n* = 50)	Group 2 (*n* = 50)	*p* value
Age (years)	41.6 ± 10.6	42 ± 10	*0.19*
Sex (F/M)	7/43	9/41	*0.29*
BMI (kg/m^2^)	43.6 ± 5.8	42.2 ± 4.8	*0.71*
Previous bariatric surgery (*n*, %)	5 (10%)	3 (6%)	*0.71*
Previous abdominal surgery (*n*, %)	10 (20%)	8 (16%)	*0.60*
Staple line reinforcement/oversewing	2 (4%)	1 (2%)	*1*
Patients with BMI > 50	6 (12%)	3 (6%)	*0.29*

Group 1 = LSG cases from 1 to 50; Group 2 = LSG cases from 51 to 100

Italic values indicate statistically significant *p* values (*p* < 0.05)

### Weight loss

**Fig. 1:**
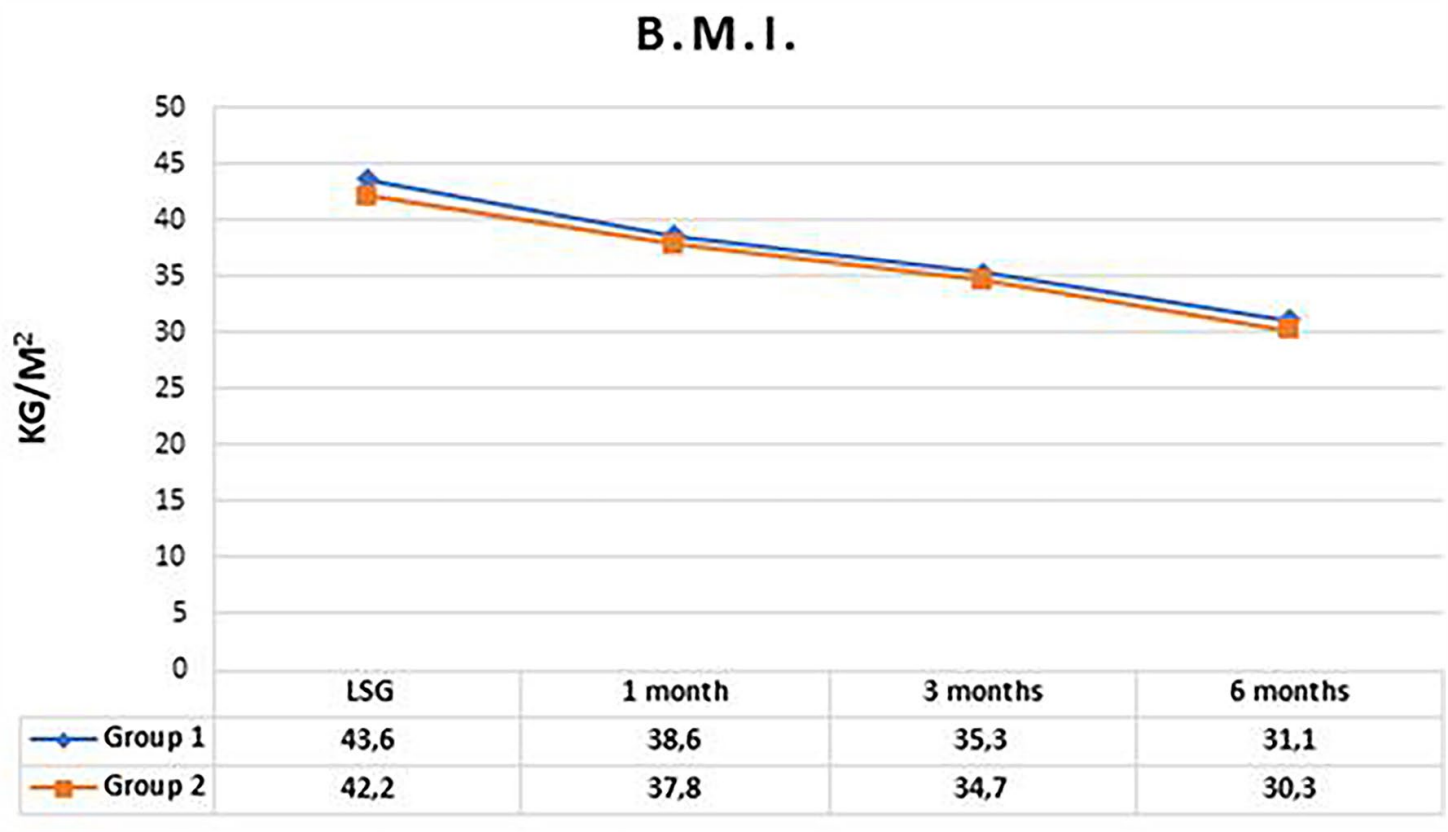
Trend of BMI in the two groups in the first 6 months. *p* value was 0.41, 0.58 and 0.48 respectively

**Fig. 2:**
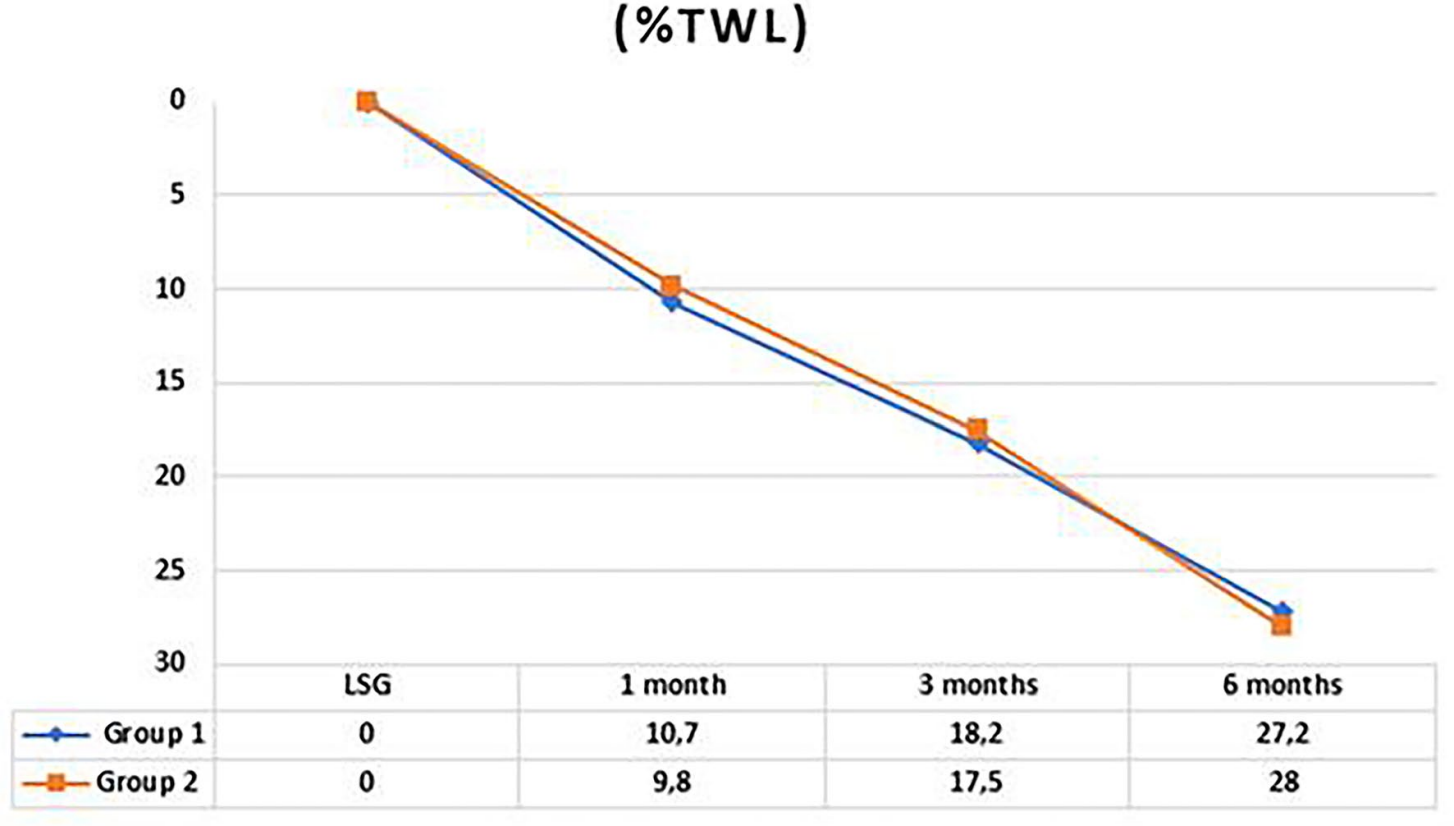
Trend of %TWL in the two groups in the first 6 months. *p* value was 0.36, 0.57 and 0.73 respectively

Mean BMI at 1, 3 and 6 months after LSG was 38.2 ± 4.9, 35 ± 4.9 and 30 ± 6 respectively. Percentage of total weight loss (%TWL) was 10.3 ± 4.5 after 1 month, 17.9 ± 6.1 at 3 months and 27.6 ± 11.2 after 6 months. No statistical difference in BMI and %TWL was found between the two groups at any time of follow-up (Figs. 1, 2). Follow-up rate at 1, 3 and 6 months were 100%, 100% and 98% in group 1 and 100%, 96% and 82% in group 2.

### Complications and comparison with global benchmarks

Mean operative time was 92.1 ± 19.3 min and hospital stay was 3.4 ± 0.6 days as per our standard protocol of discharge. Four patients had postoperative bleeding requiring transfusion and one subjects was readmitted due to hypokalaemia caused by induced vomit. Only one case of staple line leak was registered in the first 50 cases (group 1) and was successfully treated with oesophageal stenting. Uneventful postoperative course was recorded in 93% of patients. Comparison between two groups shows a significant reduction in hospital stay and operative time after the first 50 LSGs (*p* < 0.05, Tables 2 and 3). As reported in Tables 2 and 3, all benchmark values, except for bleeding, were satisfied in group 2.

**Table 2:** Comparison of peri-operative complications in the two groups with global benchmarks

Perioperative complications	Benchmark cutoffs (75th Percentile)	Group 1	Group 2	*p* value
Operation duration (min)	90	97.1 ± 21	87.1 ± 16	*0.009*
Conversion to open	0%	0	0	*1*
Intraoperative blood transfusion	0%	0	0	*1*
Postoperative blood transfusion	1.3%	(3) 6%	(1) 2%	*0.6*
Postoperative ICU admission	0%	(1) 2%	0%	*1*
ICU stay in patients admitted to ICU (days)	4	2	0	*n/a*
Hospital stay	3	3.6 ± 0.67	3.3 ± 0.5	*0.03*

Italic values indicate statistically significant *p* values (*p* < 0.05)

**Table 3:** Comparison of postoperative complications (< 90 days) in the two groups with global benchmarks

Perioperative complications until 90 days	Benchmark cutoffs (75th Percentile)	Group 1	Group 2	*p* value
Uneventful postoperative course	> 88%	(44) 88%	(49) 98%	*0.11*
Readmission	< 5.5%	1(2%)	(0) 0%	*1*
Reoperation	< 3%	0%	0%	*1*
Any complication	< 12%	(6) 12%	1 (2%)	*0.11*
Complication grade > IIIa	< 5.5%	0%	0%	*1*
Mortality	0%	0%	0%	*1*
Staple line leak	< 0.15%	1 (2%)	(0) 0%	*1*
Dysphagia/Stenosis of the gastric tube	< 0.27%	0%	0%	*1*
Postoperative bleeding	< 1.7%	(3) 6%	(1) 2%	*0.6*
Small bowel obstruction	0%	0%	0%	*1*
Wound infection	0%	0%	0%	*1*

Italic values indicate statistically significant *p* values (*p* < 0.05)

Cases requiring supervision were 7 (14%) in group 1 and 2 (4%) in group 2 (*p *< 0.15).

### CUSUM analysis of the learning curve

**Fig. 3:**
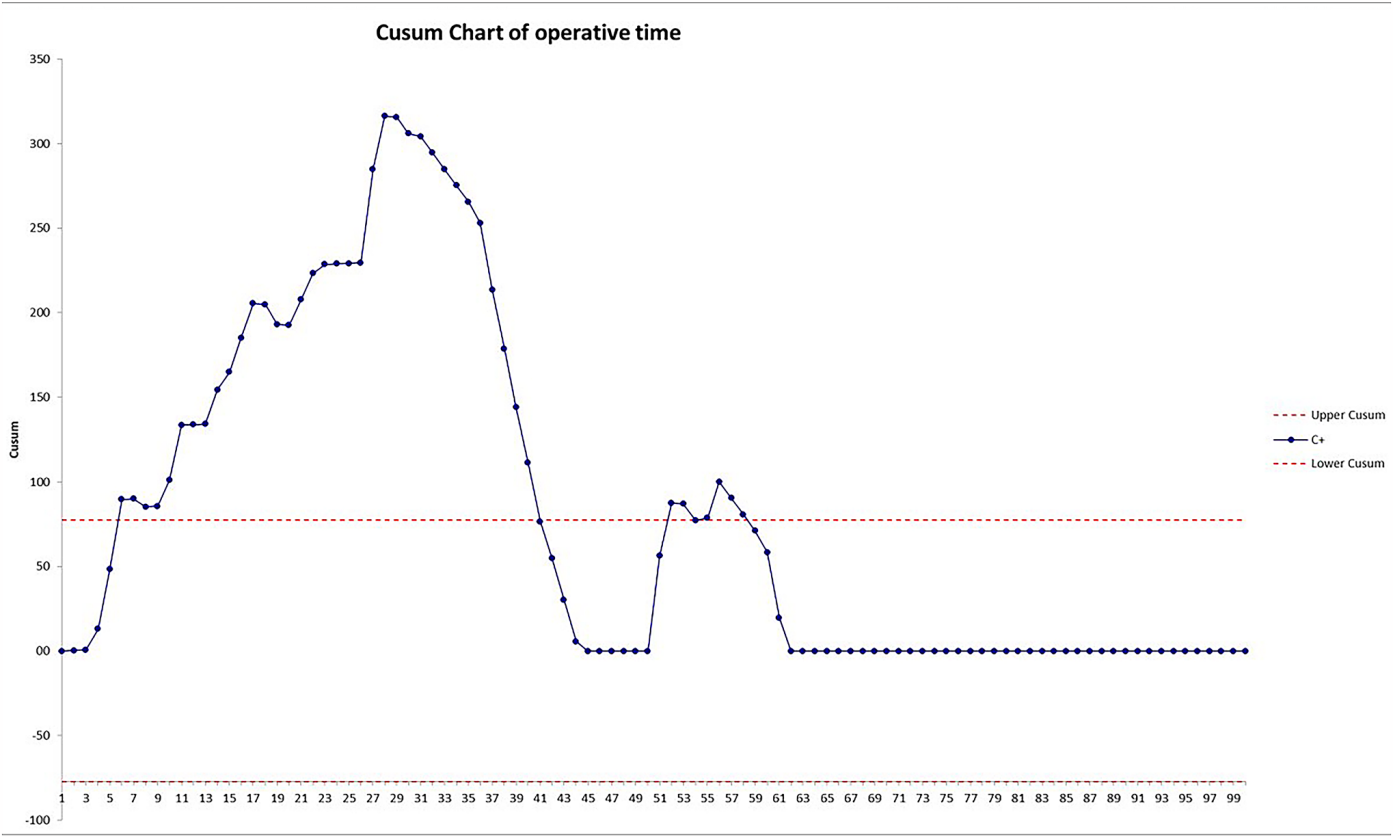
CUSUM curve of operative time

CUSUM graph of the operative time (Fig. 3) runs above the predetermined limit till the 40th cases but reaches the plateau after the 62nd operation.

**Fig. 4:**
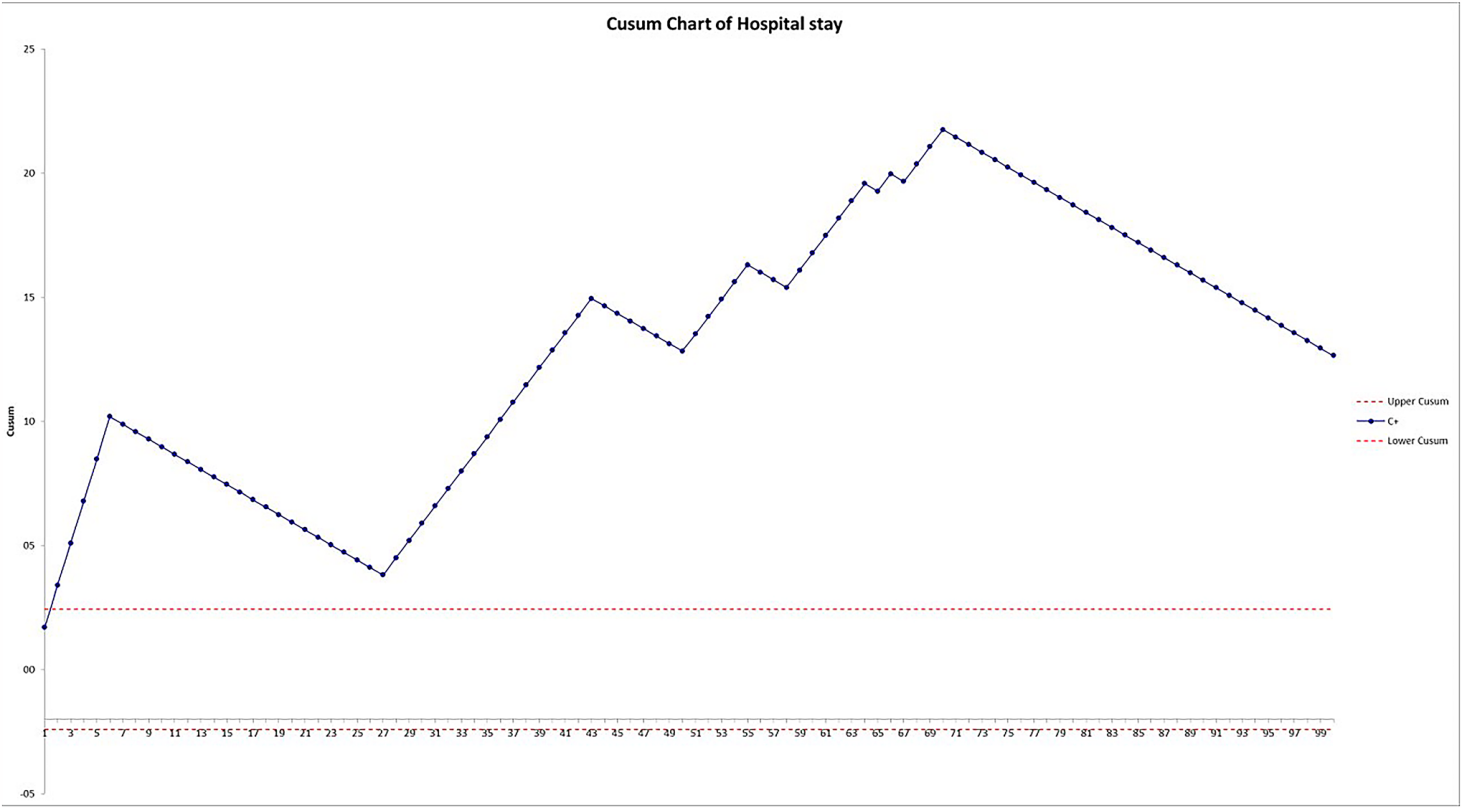
CUSUM curve of hospital stay

CUSUM graph of the hospital stay (Fig. 4) runs above the accepted limit (due to our protocol) but there is a significant slope after the 70th case.

## Discussion

Training in surgery is a continuous journey that never really ends. However, optimal operative performance can be achieved for every intervention after a certain number of procedures. There are two main methods to determine whether adequate expertise has been reached: one is to compare perioperative outcomes with international standard values; the second is to statistically and graphically analyse results with the CUSUM calculation. A recent article has demonstrated that global benchmarks [12] are useful indicators for the learning curve of the RYGB and our experience suggest that those values are applicable also for LSG.

Previous experiences have demonstrated that perioperative outcomes of laparoscopic gastrectomies for cancer [20] or for bariatric purpose^11^ significantly improve after the first 100 cases. In a study by Zachariah et al. [21], post-operative morbidity after LSG significantly decreased after the first 50 cases, while Prevot et al. [22] showed a significant difference in terms of operative time and weight loss after 28 cases.

On the contrary, other papers [23, 24] have reported that a longer case series (> 500 procedures) is necessary for RYGB. Current training of the American Society for Metabolic and Bariatric Surgery (ASMBS) requires fellows to participate in at least 100 weight-loss operations, of which at least 50 should be gastric bypass interventions [25].

Nevertheless, many factors may influence peri-operative outcomes of bariatric surgery such as previous non-bariatric surgical experience, individual skills, careful selection of patients, adequate mentoring by a senior tutor and the support of a multidisciplinary team of a high-volume centre.

Several articles have indeed proved that both LSG and RYGB can be safely performed in structured teaching program by trainee in an early stage of surgical education without untoward consequences for the patient [26, 27].

Despite these enthusiastic reports, it is undeniable that bariatric surgery required advanced laparoscopic skills, without which worrisome complications such as staple line leak, bleeding or stenosis may occur [28–32]. A recent prospective study involving 50 general surgery residency programs in the United States showed that meaningful autonomy for LSG was achieved only by 45% of the trainees in the fifth postgraduate year [33].

Our experience shows that, even if outcomes of first cases of a newly trained surgeon satisfy most benchmark cut-offs, CUSUM analyses demonstrates that a longer series of procedures is needed to reach the plateau of the learning curve. Indeed, all benchmark criteria were met in group 2, except for bleeding, but on this matter, we should consider that all postoperative haemorrhages were resolved without reoperation.

Significant shorter hospital stay was recorded in group 2 and CUSUM graph shows a plateau after the 70th case. Surgeon’s confidence could be the reason of this earlier discharge rather than his proficiency; however, this further demonstrates that at least 50 cases are needed to become confident with LSG.

As described above, our discharge protocol provides liquid diet on postoperative day 3, therefore shorter length of stay cannot be achieved in our department. We are well aware that there is a current discussion on the safety of LSG as a day case surgery [34], but this management of bariatric surgery does not apply to our hospital due to the absence of an accident and emergency unit.

Our data appear even more impressive if we take into account that these global cut-offs were defined including only patients without previous abdominal surgery and excluding superobese subjects, while we did not adopt these safety criteria.

Correct surgical technique is also important to obtain satisfactory weight loss; there is a consensus [35] that sleeve should be fashioned over an orogastric tube of at least 36 Fr starting within 4–5 cm from the pylorus to avoid leaving behind a large antrum. Subsequently, a large sleeve may be the main cause of insufficient weight loss. Since most of patients in group 2 have undergone surgery less than a year before this paper, we have focused on short term weight loss, which is an important predictor of long-term results [36]. Percentage of total weight loss was greater than 25% after 6 months without any difference between the two groups, meaning that the stomach was correctly shaped also in the first cases.

No information was collected on obesity related diseases because six months are not enough to document a significant improvement of these conditions and the duration of remission cannot be predicted with such a short period of time.

## Conclusion

Laparoscopic sleeve gastrectomy (LSG) confirms to be a feasible and effective procedure, which can be performed by newly trained surgeons proctored by senior tutors. However, perioperative outcomes within global benchmarks were achieved only after 50 consecutive operations and few more cases may be required to reach the plateau of the learning curve for operative time and hospital stay.
